# Even if they are not aware of it, general practitioners improve well-being in their adolescent patients

**DOI:** 10.1080/13814788.2017.1346077

**Published:** 2017-07-17

**Authors:** Benoit V. Tudrej, Anne-Laure Heintz, Michaela B. Rehman, Daniel Marcelli, Pierre Ingrand, Philippe Binder

**Affiliations:** ^a^ Department of General Practice, Université de Poitiers UFR Medecine et PharmaciePoitiersFrance; ^b^ Medical Ethics and Legal Medicine Laboratory, Université Paris Descartes ParisÎle-de-FranceFrance; ^c^ ADOC Group (Adolescents and Risk Behaviours), AssociationLussantFrance; ^d^ Department of Cardiology, Centre Hospitalier Universitaire de PoitiersPoitiersFrance; ^e^ University Clinic of Child and Adolescent Psychiatry, CHU Poitiers and Centre Hospitalier Henri Laborit, Faculty of MedicinePoitiersFrance; ^f^ Department of Epidemiology & Biostatistics, INSERM CIC-1402, Faculty of MedicinePoitiersFrance

**Keywords:** Adolescent, well-being, general practitioner, consultation, primary care, patient–physician relationship

## Abstract

**Background:** Most adolescents consult their general practitioner (GP) for common reasons, somatic or administrative but many of them have hidden feelings of distress.

**Objectives:** To assess the immediate impact of ‘ordinary’ consultations on feelings of distress among adolescents and to compare adolescents experiencing difficulties (D) to those with no difficulties (N). To analyse how accurately GPs assess the impact of their consultation on adolescents’ feelings.

**Methods:** GPs were randomly selected from two non-contiguous French administrative areas between April and June 2006. Fifty-three GPs gave two questionnaires to the first 10 to 15 adolescents aged 12 to 20 seen in consultation. One questionnaire was issued before the consultation and the other one afterwards. Adolescents had to position themselves about different aspects of well-being and say where they would seek help if they had problems. A GP questionnaire assessed how well they estimated their impact on the adolescent’s feeling of well-being.

**Results:** Six hundred and sixty-five adolescents were assessed. They reported feeling better about their health, being able to talk, having someone to talk to or to confide in and on feeling understood. The D group (*n* = 147) felt significantly better compared to the N group (*n* = 518). GPs tended to underestimate this improvement, especially regarding adolescents in the D group feeling better about their health.

**Conclusions:** Consulting a GP generates increased well-being among adolescents, especially for those experiencing difficulties. GPs tend to underestimate the positive impact they may have. Further studies are needed to explore if this benefit is permanent over time.

Key messages

- Consulting a GP has an important positive psychological impact on adolescents and especially so for those who experience distress.
- GP tend to underestimate the improvement adolescents feel after the consultation.
- GPs do not correctly assess adolescents’ perceptions of their health status, in particular in adolescents having trouble.

## Introduction

Many adolescents experience difficulties and psychological issues [1]. However, these disturbances are difficult to describe in nature and intensity [2]. On the General Health Questionnaire 12 (GHQ 12), a quarter of young people complain of psychological upheaval, and half report distress or impaired well-being [3]. It is of major importance to detect these issues because they are correlated with risk-prone and acting-out behaviour [4].

Adolescents find it difficult talk to a doctor about difficulties they are facing. This may be because they think that GPs only deal with somatic problems [5], cannot provide ‘talking’ therapy, and may be dismissive of psychological distress [6]. Adolescents consult a GP for a psychological reason in no more than 8% of cases [7]. Identifying mental issues is even more complicated in these adolescents because they do not tend to seek specific assistance [8–10]. They often report trouble communicating with adults [11].

Conversely, general practitioners (GPs) do not always detect which adolescents are in distress and sometimes feel unable to help [12,13]. But youth suicide could be prevented by earlier recognition and treatment of psychological distress [14]. Thanks to their communication skills alone, consulting a GP seems to help adolescents talk about personal difficulties [13,15,16]. Some studies have analysed what makes the consultation more beneficial for patients [17]. Others have assessed how the GP’s consulting style affects patient satisfaction. However, to our knowledge, no study has analysed if the consultation can affect adolescents’ feelings and how GPs perceive the benefit of the consultation. 

This quantitative study aimed to answer two questions: (a) Do consultations with a GP improve adolescents’ well-being; and, are there differences between adolescents experiencing distress or not? (b) Do GPs perceive their impact on adolescents’ well-being accurately? Our hypotheses are that (a) a consultation with a GP does not have the same impact on adolescents experiencing greater or lesser distress; and (b) that GPs underestimate the benefit of their consultations on adolescents’ well-being.

## Methods

### Study design

This study was performed in two non-contiguous French administrative areas of the French Region ‘Poitou-Charentes’ between April and June 2006. The population is representative of the overall French population with no ethnic or cultural specificity. In the application of French law, this study did not require approval by an ethics committee. This paper is the third article from the same original study (Box 1).

Box 1 Previous works

Three different articles were written from the same original study the current paper was part of.

The first article showed an improvement in adolescents’ feelings after consultation. This improvement is independent of the doctor’s experience in adolescents’ issues [24]. The second article showed that a third party does not influence how adolescents talk to their GPs [22]. The current paper explores the fact that adolescents experiencing difficulties feel better than adolescents without difficulties after an ‘ordinary’ consultation. It took 10 years to submit it for publication because one of the investigators gave up on this work. However, we thought it was relevant to share this unknown data.

### Selection of GPs and adolescents

The investigators were GPs in private practice. They were randomly selected from a free online doctor directory and individually contacted by investigators. The solicitation ended when 30 GPs accepted to take part in the study in each administrative area. After signing the agreement form, GPs were to give every consecutive adolescent aged 12 to 20 years seen in consultation for whatever reason the opportunity to take part in the study. They were to include between 10 and 15 adolescents.

### Measurements

#### Questionnaires

Each consultation generated three questionnaires. Whether accompanied or not, the adolescents completed two questionnaires confidentially; one before the consultation and another one afterwards. The GPs completed a questionnaire after each consultation. Three experts designed the questionnaires: a GP (PB), a professor in paediatric psychiatry (DM), and a professor of public health (PI). In a previous study, 29 adolescents had tested the questionnaires in consultation with 10 GPs and the wording was optimized. There were 10 items in the ‘Adolescent before consultation’ questionnaire: age (in years); three statements using multiple-choice answers about gender (male/female), reason for consulting (somatic, psychological, administrative or preventive reason) and having multiple worries (no other worry, other worry and happy to talk about it, other worry but do not want to talk about it); and five statements using visual analogue scales (VAS).

#### Visual Analogue Scales

Adolescents had to position themselves in relation to how they perceived their health status, to what extent they had someone they could talk about problems with, someone to confide in if they had problems and someone they felt could understand them. Another VAS about music whose answer we assumed did not change during the consultation, tested the level of VAS reliability.

The VAS were composed of a 10 cm horizontal line with small perpendicular graduation marks, 3 mm apart so that their number was big enough to prevent visual tracking. A light green cone increased from the negative answer (a point) to the positive answer (12.5 cm). At each extremity, text explained the ideas on which the adolescents and GPs had to position themselves (Box 2).

Box 2 Information available above and at extremities of visual analogue scale used in questionnaire Q1, Q2 and Q3.

**Table:** 

Q1: Adolescent questionnaire before consultation		Q2: Adolescent questionnaire after consultation		Q3: GP questionnaire after consultation	
**Health status**					
At the moment, …				At the moment, I think that this adolescent…	
I feel in very poor health		I feel in very good health		feels he/she is in very poor health	feels he/she is in very good health
**Can talk to**					
At the moment, if I have a problem or a worry, …				At the moment, if the adolescent has a problem or a worry, I think he/she …	
I will be able to talk about it very easily		I will not be able to talk about it at all		will find it easy to talk about it	will be unable to talk about it
**Someone to confide in**					
At the moment, if I have a problem or a worry, …				At the moment, if the adolescent has a problem or a worry, I think he/she …	
I really have no-one I can confide in		I always have someone I can confide in		has really no-one to confide in	always has someone to confide in
**Feeling understood**					
Generally, if I talk about my problems or my worries, …		During this consultation, I felt that…		During this consultation I think this adolescent did not feel that he/she…	
I do not feel understood at all	I feel perfectly understood	I was not at all understood	I was perfectly understood	was at all understood	felt perfectly well understood
**Liking music**					
Generally, …					
I really like to listen to music		I really do not like to listen to music			
**Expectations**					
		This consultation lived up to my expectations		I think this consultation lived up to this adolescent's expectations.	
		No, not at all	Yes, completely	No, not at all	Yes, completely

The VAS was oriented randomly from the right or the left to focus attention, and the subject was asked to position him-/herself between the two extremities. The distributions did not differ between these VAS and alternative approaches (numeric input scales, radio button scale, VAS with or without feedback) [18].

The ‘Adolescent after consultation’ questionnaire included the same five VAS and an extra VAS assessing how the consultations met their expectations. The GP’s questionnaire analysed the GP’s perception of the adolescent’s thoughts and feelings with a VAS. The items were the same as in the adolescent ‘after consultation’ questionnaire except for the music question (Box 2).

#### Procedure

The adolescents were informed about the study by the medical secretary of the GP and by a poster in the waiting room. Before the consultation, GPs explained the procedure and asked for the informed consent of the first 10 to 15 consecutive adolescents whatever their reason for consulting. GPs submitted the questionnaire and the adolescents filled it in (Q1) in the waiting room. The adolescent gave the questionnaire in a sealed envelope directly to the GP. After the consultation, adolescents and GPs filled in the two others questionnaires (Q2, Q3), separately. A number for the consultation and a number for the GP linked the three questionnaires. The database was collected and constructed by a public health professor (PI).

### Statistical analysis

Adolescents were split into two groups according to their answers to the first questionnaire: experiencing difficulties (D) or with no difficulties (N). To be included in the D group, a positive answer to at least one of those two items were required: ‘I am seeing the doctor today first and foremost for psychological reasons’ and ‘I have worries other than the reason for which I am consulting today’ (irrespective of whether the subject wanted to talk about them). The N group included all other adolescents.

No data was eliminated from the analyses, because there was no significant variation or difference between groups on the music question.

The positions of the marks on the VAS were measured by 200 mm ruler, deriving a value between 0 and 100. Data was captured in an EpiInfo6 database. A second operator repeated the measures independently.

Descriptive analysis of the answers and the changes in feelings before and after the consultation were performed in each group. The χ^2^ test for proportions and the non-parametric Mann–Whitney test for scales were used for comparisons between groups using SAS/STAT software. Matched comparisons (before versus after consultation, adolescent versus GP) were performed using the paired Wilcoxon test. Between-groups comparisons of evolutions were analysed with covariance analyses adjusted for age and gender.

An estimated sample size of 600 consultations was required to provide 90% power and to detect a minimum 10-point difference on the 100-point scale (as used in pain VAS in which 10 mm is the smallest clinically relevant difference) with a standard deviation ≤30 mm in the comparison between adolescents experiencing difficulties and the others, if the prevalence of difficulties was 20% [19].

## Results

### Description of GPs

Fifty-three GPs (17 women, 36 men) aged 32 to 60 years (mean age 45.8 years) took part. There were more GPs working in urban areas (Table 1).

**Table 1.** Demographic data of adolescents. One adolescent did not fill in his gender. We excluded this result in the gender comparisons, but we included it in the others analyses.

	All adolescent patients *n* = 664	No difficulties (N) group *n* = 517	Difficulties (D) group *n* = 147	*P*
F (%)	395 (59.5)	290 (56.0)	105 (71.4)	0.0008^a^
Age: mean (min–max)	16.0 (12–20)	15.9 (12–20)	16.3 (12–20)	0.026^a^

^a^ χ^2^ test.

### Description of adolescents

**Figure 1.**
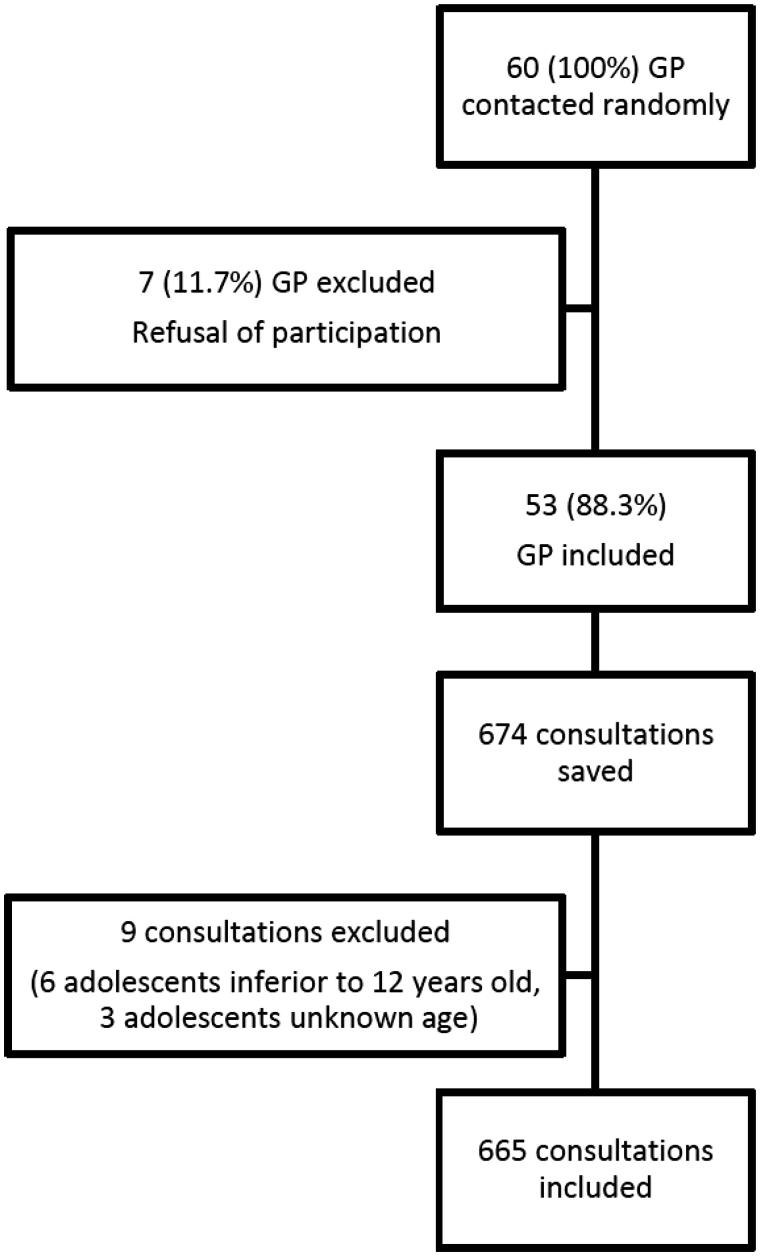
Flow chart.

Nine consultations had to be excluded: six patients were under 12 and three adolescents did not notify their age. One adolescent did not disclose his gender. Therefore, we excluded this result in gender comparisons, but included it in the other analyses (Figure 1). 

Overall, 665 adolescents were analysed, predominantly women (59.5%) and 64% were accompanied: 80% by their mother, 10% by their father (Table 2).

**Table 2.** Variations in adolescents’ feelings before and after consultation, according to groups experiencing difficulties (D) versus not experiencing difficulties (N).

	Before consultation			After consultation			Evolution of the groups				
	N	D		N	D		N	Intra group	D	Intra group	Between groups age/sex adjusted
	*n* = 518	*n* = 147	*P*	*n* = 518	*n* = 147	*P*	*n* = 518	*P*	*n* = 147	*P*	*P*
Feeling good health status	69 (26) [66; 71]	54 (27) [50; 59]	<0.0001^a^	74 (22) [72; 76]	66 (23) [63; 70]	0.0004^a^	5 (19 [4; 7])	<0.0001^b^	12 (24) [8; 16]	<0.0001^b^	0.0008^b^
Being able to talk about problems	66 (26) [64; 69]	52 (28) [47; 56]	<0.0001^a^	74 (23) [72; 76]	68 (24) [64; 72]	0.0027^a^	8 (20) [6; 9]	<0.0001^b^	16 (25) [12; 20]	<0.0001^b^	<0.0001^b^
Having someone to confide in	81 (21) [79; 83]	67 (28) [63; 72]	<0.0001^a^	82 (19) [81; 84]	75 (22) [71; 79]	<0.0001^a^	1 (17) [0; 3]	0.42^b^	8 (19) [4; 11]	<0.0001^b^	0.0003^b^
Feeling understood	70 (22) [68; 72]	54 (27) [49; 58]	<0.0001^a^	85 (16) [83; 86]	77 (21) [73; 80]	<0.0001^a^	15 (21) [13; 16]	<0.0001^b^	23 (23) [19; 27]	<0.0001^b^	<0.0001^b^
Liking music	88 (18) [87; 90]	88 (18) [85; 91]	0.44^a^	89 (16) [88; 90]	89 (16) [86; 92]	0.87^a^	1 (10) [0; 2]	0.60^b^	1 (13) [-1; 3]	0.46^b^	0.50^b^

N = no difficulties group; D = difficulties group. Results on visual analogue scales ranging between 0 and 100 are presented as mean (standard deviation) and [95% confidence limits].

The higher VAS score is considered as ‘better’ score.

^a^ Non-parametric Mann–Whitney test.

^b^ Paired Wilcoxon test.

Only 8% of girls and 4% of boys consulted for psychological reasons. Among those consulting for administrative or somatic reasons, 17% notified they had ‘worries’ other than the reason of their visit. In the D group, adolescents were predominantly female and slightly older than in the N group. These small differences did not affect the significance of the statistical results, as can be seen from comparisons adjusted for age and gender.

### Results for the adolescent patients

Both groups of adolescents reported that they felt better about the four feelings (Table 2). In the D group, the improvement was significantly greater on the four feelings (Table 2). The N group scored significantly higher on the feeling that the consultation met their expectations: 86 (±16) versus 76 (±22) (*P* <0.0001). The D group reported a poorer health status and greater difficulty talking about their problems; they were more likely to have nobody to confide in, and they were less likely to feel understood when they did talk about their problems (Table 2).

### GPs assessment of adolescents’ well-being

GPs underestimated how adolescents felt about their health status and this was significantly greater in the D group (*P* = 0.016). GPs also thought adolescents were less likely to have someone to confide in and to be understood than they were without significant difference between groups. GPs underestimated the fact that their consultation met adolescents’ expectations without significant difference between groups. No specificity linked to age was found for any of these results. The detailed comparison between GPs’ representations and adolescents’ feelings are shown in Table 3.

**Table 3.** Differences between general practitioners’ representations and adolescents’ feelings.

		N *n* = 518	Intra group *P*	D *n* = 147	Intra group *P*	Inter-group age/sex adjusted *P*
Feeling good health status						
Adolescent	I feel in very poor health//in very good health	–3 (24) [–5; –1]	0.0025^a^	–9 (26) [–13; –4]	<0.0001^a^	0.016^a^
GP	I think that this adolescent feels he/she is in very poor//very good/health					
Being able to talk about problems						
Adolescent	I can talk about any problems I may have	2 (25) [–1; +4]	0.51^a^	4 (26) [0; +9]	0.080^a^	0.35^a^
GP	I think that now if this teenager has a problem he/she will be able to talk about it					
Having someone to confide in						
Adolescent	I have someone to confide in if I have a problem	–5 (22) [–7; –3]	<0.0001^a^	–3 (24) [–7; +1]	0.078^a^	0.27^a^
GP	I think that now if this teenager has a problem or worry he/she will be able to confide in someone					
Feeling understood						
Adolescent	During this consultation, I felt I was understood	–6 (19) [–8; –4]	<0.0001^a^	–5 (21) [–8; –1]	0.0005^a^	0.46^a^
GP	During this consultation, I think the teenager felt he/she was understood					
Expectations						
Adolescent	This consultation lived up to my expectations	–7 (20) [–9; –5]	<0.0001^a^	–6 (19) [–9; –3]	0.0001^a^	0.52^a^
GP	I think that this consultation lived up to this teenager's expectations.					

N = no difficulties group; D = difficulties group. Figures are means (standard deviations) and [95% confidence limits] of differences between paired VAS ratings of the adolescent and the GP. A negative mean value indicates a lower rating by the GP than by the adolescent himself.

^a^ Paired Wilcoxon test.

## Discussion

### Main findings

In this study including 665 French adolescents visiting their GP, all adolescents reported feeling an improvement in their physical and psychological health after the consultation. This effect was more pronounced in the D group. GPs tended to underestimate this improvement and did not assess the adolescents’ feeling on their health status accurately, especially in the D group.

### Strengths and limitations

Our results are reliable, thanks to the number of consultations and the participation of the vast majority of the randomly selected GPs.

The main limitation of this study is the method used for splitting the adolescents into two groups. We did not use any standard instrument to explore the mental status, such as the GHQ-12, the KADS or the MADRS [20]. Rather than establishing an objective psychological diagnosis, the aim was to focus on the change in state of mind of worried adolescents, whether or not they put their worries into words, during a consultation. We analysed their state of mind mainly via their representations of where to turn for help if they had problems.

The choice of age range is due to French legislation for adolescent hospital wings [12–18]. We added two years because many youths live with their parents until the age of 20. However, age-adjusted analyses showed no significant differences.

The reliability of 10-year-old data can be discussed. However, GP–adolescent relation issues encountered by adolescents in France are not linked to time, because nothing has changed in GP consultations: face-to-face consultations, payments, perception of professional secret.

We preferred to use VAS scales rather that Likert scales because the result after consultation is not influenced by the numerical value chosen in the pre-consultation questionnaire. Furthermore, scales anchored with terms, labels or numbers induce floor or ceiling effects [21]. This could make GPs’ and adolescents’ comparisons less reliable. As no VAS type can be considered a gold standard to measure subjective symptoms, this bias cannot be estimated itself [21].

Filling in the pre-consultation questionnaire in the waiting room is questionable because the confidentiality is not perfect and the companion may have been present when adolescents filled out the questionnaire. However, literature showed that most often adolescents liked being accompanied, and that this did not prevent them from talking about their worries [22]. One could explain this by the reassuring role of parental presence and because confidentiality is only a concern for a minority of adolescents and mainly for the older ones [23]. However, it seemed the best place possible to ensure confidentiality and distance from the GP and secretary.

GPs submitted the questionnaire to adolescents themselves, so this could favour the perception of the usefulness of the consultation. This could bias the results, but no more than in any observational study.

The results are also limited by the short time-span between the two measures, 15 to 20 minutes, and the possible influence of the GP close at hand. Since the second questionnaire was completed immediately after the consultation, the positive effects reported by the adolescents could be due to the immediate feelings of friendliness, which might wane back home. However, this effect was more marked in the D group, emphasizing the value of an ‘ordinary’ consultation for these subjects.

### Interpretation of the study results

Adolescents that were initially assigned to the D group had a more negative assessment of themselves in the four VAS questions. Our observations are in agreement with studies that have shown that adolescents having trouble also have communication deficits, in particular, towards adults [11].

The greater positive effects on the D group could encourage adolescents to turn to their GPs again in the future. This result is consistent with findings that adolescents turn more easily to help providers with whom they have had a positive experience [11].

GPs’ role in this improvement is debatable. This could occur with any formalized encounter with an adult. To analyse this point, a similar study is required involving another adult (teacher, hairdresser, etc.).

The fact that GPs do not assess their impact on adolescents correctly is an important message and has been already observed [16]. This could be explained by GPs overestimating problems and focusing mainly on somatic concerns.

After the two studies published from the same dataset [22,24], this third article bridges a gap in this research area. Qualitative literature shows that GPs can have a major role in detecting and helping adolescents with psychological issues, within specific protocols [25,26]. Therefore, our quantitative study has demonstrated something new: any GP consultation, for whatever reason can, in itself, improve the well-being in adolescents. An important reason for GPs disengaging in this area is the uncertainty about what to do and what is expected of them as primary care clinicians [27]. GPs often feel a sense of professional disempowerment: stemming from a lack of formal training and education about the clinical topic [28]. Our study shows that this feeling is very subjective. Indeed, GPs significantly underestimate their impact on immediate well-being in adolescents.

### Implication for clinical practice

This study should encourage GPs to examine broader issues with adolescents whatever their reason for consulting.

## Conclusions

GPs often underestimate the positive impact they have had on adolescents, who feel significantly better, especially if they have been experiencing difficulties.
